# Quantitative Molecular Analysis of Sentinel Lymph Node May Be Predictive of Axillary Node Status in Breast Cancer Classified by Molecular Subtypes

**DOI:** 10.1371/journal.pone.0058823

**Published:** 2013-03-22

**Authors:** Simonetta Buglioni, Franco Di Filippo, Irene Terrenato, Beatrice Casini, Enzo Gallo, Ferdinando Marandino, Carlo L. Maini, Rossella Pasqualoni, Claudio Botti, Simona Di Filippo, Edoardo Pescarmona, Marcella Mottolese

**Affiliations:** 1 Department of Pathology, Regina Elena National Cancer Institute, Rome, Italy; 2 Department of Surgery, Regina Elena National Cancer Institute, Rome, Italy; 3 Biostatistics, Regina Elena National Cancer Institute, Rome, Italy; 4 Department of Nuclear Medicine, Regina Elena National Cancer Institute, Rome, Italy; Health Canada, Canada

## Abstract

To determine the performance of intraoperative one-step nucleic acid amplification (OSNA) assay in detecting sentinel lymph node metastases compared to postoperative histology taking into account breast cancer molecular classification and to evaluate whether the level of cytokeratin 19 mRNA copy number may be useful in predicting the likelihood of a positive axillary lymph node dissection. OSNA assay was performed in a prospective series of 903 consecutive sentinel lymph nodes from 709 breast cancer patients using 2 alternate slices of each sentinel lymph node. The remaining 2 slices were investigated by histology. Cytokeratin 19 mRNA copy number, which distinguishes negative cases (<250 copies), micrometastases (+, ≥250≤5000 copies) and macrometastases (++, >5000 copies), was compared to axillary lymph node dissection status and to the biological tumor profile. Concordance between OSNA and histopathology was 95%, specificity 95% and sensitivity 93%. Multiple Corresponce Analysis and logistic regression evidenced that positive axillary lymph node dissection was significantly associated with a higher cytokeratin 19 mRNA copy number (>5000; p<0.0001), HER2 subtype (p = 0.007) and lymphovascular invasion (p<0.0001). Conversely, breast cancer patients with cytokeratin 19 mRNA copy number <2000 mostly presented a luminal subtype and a negative axillary lymph node dissection. We confirmed that OSNA assay can provide standardized and reproducible results and that it represents a fast and quantitative tool for intraoperative evaluation of sentinel lymph node. Omission of axillary lymph node dissection could be proposed in patients presenting a sentinel lymph node with a cytokeratin 19 mRNA copy number <2000 and a Luminal tumor phenotype.

## Introduction

Sentinel lymph node (SLN) biopsy is a highly accurate predictor of overall axillary status and in the last 15 years has become the standard method in breast cancer (BC) patients who are clinically negative for lymph node [1], [2]. In cases of negative SLN, patients can safely avoid axillary lymph node dissection (ALND), thus preventing the associated morbidity [3]. Nevertheless, adequate diagnostic standardization has yet to be achieved and the protocols for the histological evaluation of the SLN are highly variable between centers. Application of multilevel sectioning and immunohistochemistry (IHC) have increased the accuracy of metastatic deposit detection by up to 25% compared to standard morphological analysis. Despite this, the lack of well standardized guidelines leads to great difficulties in comparing results among different diagnostic approaches, particularly when the SLN is infiltrated by micrometastasis (<0.2 mm) or isolated tumor cells (ITC) [4]. In addition, conventional histological methods may provide conclusive results only postoperatively. This is a significant limit since ideal management in BC patients with positive SLN should include the ALND during the same operation in order to avoid both the cost and the burden of a second surgery. Aimed to more rapidly assess the SLN status, different intraoperative diagnostic procedures, such as frozen sections or imprint cytology, are currently used. Although the latter procedures are low-cost and display a high specificity (95–100%), they are quite variable and not very sensitive (58–87%) compared to postoperative histology, especially in detecting micrometastases or ITC [5]–[7]. As a consequence, there is an urgent need for alternative, highly standardized and reproducible methods to be applied intraoperatively. A semi-automated molecular method called the one-step nucleic acid amplification (OSNA) assay has recently been made available. This procedure allows a rapid intraoperative evaluation of SLN status. The method, based on the direct quantification of the cytokeratin 19 (CK19) mRNA in about 30–40 minutes provides quantitative results which are related to the size of the metastases [8]. As highlighted in the last St Gallen Conference [9], BC should be considered a heterogeneous disease in which different subtypes may be detected by genetic array testing [10]–[12] and by IHC as surrogate. In fact, according to the expression of a few protein biomarkers, BC can be divided into four main subtypes with distinct behavior in terms of prognosis and response to therapy: Luminal A (LA) and Luminal B (LB), both estrogen (ER) and progesterone (PgR) receptors positive, but characterized by a low and high proliferation index respectively, HER2 subtype defined by overexpression/amplification of the HER2 gene and Triple Negative/basal-like (TN) lacking ER, PgR and HER2 expression. To date, there are limited published data concerning the evaluation of SLN status by OSNA in BC stratified by molecular subtypes. In February 2008 we introduced the OSNA method in our Institute with the clinical aim to provide standardized results intraoperatively. The aims of this observational prospective study were twofold: (a) to compare the performance of the intraoperative OSNA assay with conventional postoperative histological procedures in 903 SLNs sampled from 709 early BC patients (b) to determine whether the CK19 mRNA copy number in the SLN may predict the risk of a positive ALND within the different BC molecular subtypes.

## Materials and Methods

### Study Population

The study was reviewed and approved by the ethics committee of the Regina Elena National Cancer Institute (Prot. CE/913/10). The study was conducted from February 2008 to December 2010, prospectively testing 903 fresh SLNs sampled from 709 consecutive patients bearing a tumor with a maximum diameter of 3 cm or less and with clinically non palpable axillary lymph nodes. Patients with locally advanced BC (T3–T4), or with a previous diagnosis of another type of carcinoma, previous breast or axillary surgery or receiving neoadjuvant therapy were excluded from the study. Patients were subjected to modified radical mastectomy or breast-conserving surgery (quadrantectomy) and, in cases where the OSNA assay were positive, ALND was performed in the same operative session of the SLN biopsy. In our consecutive series of patients we tested 62 in situ (54 intraductal and 8 intralobular) and 647 invasive (596 ductal, 43 lobular and 8 other) BC. Tumors were graded according to Bloom and Richardson and staged according to the Unione Internationale Contre le Cancer tumor-node-metastasis (TNM) system criteria [13]. The pathological characteristics of the 647 invasive BC are shown in Table 1. In the subset of invasive BC, the OSNA results were analyzed within the four different molecular subtypes: LA (ER/PgR+, HER2− and Ki-67≤15%), LB (ER/PgR+, HER2− and Ki-67>15%), TN (ER/PgR and HER2−), and HS (ER/PgR−/+ and HER2+). A written informed consent was obtained from all patients before surgical procedures.

**Table 1:** Clinico-pathological characteristics of the 647 invasive breast carcinomas.

Characteristics	Number of cases	%
**Number of patients**	**647**	
Median age (range)	55 (26–83)	/
**Histotype**		
Ductal	596	92
Lobular	43	6.6
Other	8	1.2
**Grading**		
G1	56	8.7
G2	465	71.9
G3	126	19.4
**Tumor size**		
T1a	80	12.4
T1b	122	18.8
T1c	293	45.3
T2	152	23.5
**Lymph node status**		
N0	454	70.2
N1mi	87	13.4
N1	88	13.6
N2	14	2.2
N3	4	0.6
**LVI**		
Absent	503	78
present	144	22

LVI: lymphovascular invasion.

### Sentinel Lymph Node Sampling Method

SLNs were identified using technetium 99m-labeled, nano-sized, human serum albumin colloids. To avoid any contamination during tumor manipulation, SLNs were surgically excised before the lumpectomy and sent on ice to the Pathology Department. Each SLN was weighed, measured and divided into four nearly equal slides (a, b, c, d) with a special cutting device consisting of three blades [8]. SLNs with a weight less than 50 mg were excluded from the study. SLNs with a weight of more than 600 mg were either halved or cut into several pieces, and each piece was divided into four slices. Alternate slices (a&c) were processed by the OSNA method. The remaining two slices (b&d) were fixed with neutral buffered formaldehyde and embedded in a single paraffin block for postoperative histological examination as described below.

### One-step Nucleic Acid Amplification

OSNA assay was performed according to the manufacturer’s instructions (Sysmex, Kobe, Japan). In short, slices a&c cut from the SLN was homogenized in 4 ml of the Lynorhag homogenizing buffer (Sysmex) on ice. A small aliquot was used for automated real-time amplification of CK19 mRNA via reverse transcription loop-mediated isothermal amplification (RT-LAMP) with the ready-to-use Lynoamp Kit (Sysmex) on the RD-100i (Sysmex). It was possible to analyze up to 4 SLNs in one run. The degree of amplification was detected via a by-product of the reaction, i.e. pyrophosphate. After use, the excess lysate was stored at −80°C. A CK19 mRNA copy number/µl lysate (a) less than 250 was regarded as negative (−); (b) from 250 to 5000 as positive, score +, and (c) more than 5000, score ++. The OSNA results were immediately communicated by telephone to the Surgery Department within 30–40 minutes. In case where there was a positive OSNA result, both for micrometastasis (+) and macrometastasis (++), the patient underwent ALND in the same operative session.

### Histological Work-up

Four µm thick sections were cut from the slices b and d of the SLN, stained with Haematoxylin/Eosin (H&E), and immunostained for both anti-pan cytocheratin monoclonal antibody (mAb) MNF116 (Dako, Milan, Italy) and anti-CK19 mAb RCK108 (Dako). If the initial sections were tumor positive no further sections were cut. Otherwise, additional sections at further 6 levels at an interval of 100 µm were cut and analyzed both morphologically and immunohistochemically. When the SLN was OSNA positive (+ or ++) and, subsequently, morphologically negative, the histological work-up was extended to all levels of paraffin blocks with an interval of 50 µm. Otherwise, when the SLN was OSNA negative and morphologically positive (micrometastases or macrometastases), patients subsequently underwent ALND. In addition, as a control, the OSNA assay was repeated after checking the IHC expression of CK19 on the corresponding primary tumor.

Metastatic deposits in the SLN were recorded, according to the TNM classification, as follows: a. isolated tumor cells (ITC) if their largest diameter was smaller than 0.2 mm, b. micrometastases if they were larger than 0.2 mm but not larger than 2 mm in diameter, c. macrometastases if they were larger than 2 mm in diameter. In this study, according to the TNM definition, lymph nodes presenting only ITC were considered negative [pN0 (i+)]. Axillary non-sentinel lymph nodes were routinely examined by H&E staining.

### Immunohistochemistry

The presence of metastatic cells in the slices b&d of the SLN were further evaluated by IHC using mAb anti-pancytokeratin (MNF116, Dako) and anti-CK19 (RCK108, Dako). Immunoreactions were revealed by a streptavidin-biotin enhanced immunoperoxidase technique in an automated autostainer (Bond™ Max, Menarini, Florence, Italy). Our series of primary breast tumors were tested for ER and PgR expression using mAb 6F11 (Menarini) and mAb 1A6 (Menarini) respectively, for proliferative activity using the anti Ki-67 mAb (MIB1, Dako), for HER2 overexpression using the polyclonal antibody A0485 (Dako). TN tumors were also characterized for epidermal growth factor receptor (EGFR) (Pharmdx kit, Dako) and cytokeratin 5 expression (mAb XM26, Menarini). HER2 IHC positivity was determined according to ASCO/CAP guidelines [14] and was scored as follows: 0 and 1+ negative, 2+ equivocal, and 3+ positive. ER and PgR were considered positive when >10% of the neoplastic cells showed distinct nuclear immunoreactivity, whereas Ki-67, based on the median value of our series, was regarded as high if more than 15% of the cell nuclei were immunostained. Evaluation of the IHC results, blinded to all patient data, was performed independently and in blinded manner by two investigators (SB and MM).

### Silver In Situ Hybridization

To assess HER2 gene amplification in tumors score 2+ by IHC we used a fully automated single color in situ hybridization assay based on the use of a validated silver deposition technology (SISH, Inform HER2 DNA Probe; Inform Chromosome 17 probe, Roche Tissue Diagnostic, Milan, Italy). The silver precipitation was visualized as a black dot in cell nuclei with 100× oil immersion objective. SISH results were analyzed by using a light microscope (Nikon, Eclipse 55i) equipped with a software able to capture images (Eureka Interface System). Cases were defined as amplified when SISH displayed a gene copy number >6. Polysomy 17– intended as an increased centromere 17 enumeration probe copy number – is considered to be present in BC when a mean number of 3 signals is shown.

### Statistical Analyses

#### Patient dataset

All descriptive statistics were calculated. The proper method of analysis and the tests for statistical significance depended on the variables under study. The categorical variables were reported through frequencies and percentage values, while we considered age at surgery as a continuous variable, reporting it through the median value and its range.

#### Lymph node dataset

Correlations among tests were estimated using the Cohen’s Kappa Test. This test expresses the amount of agreement (over and above the expected due to chance alone) between the two assays.

Specificity, sensitivity, negative and positive predictive value (NPV and PPV), and the 95% confidence interval (CI) of the OSNA assay were estimated considering histology as the gold standard. The correlation between the size of intranodal metastasis (expressed in squared centimeters) evaluated by morphology and the copy number of CK19 mRNA evaluated by OSNA was determined using the non-parametric Spearman correlation coefficient. A p-value <0.05 was considered to be statistically significant.

#### Multiple correspondence analysis

Multiple correspondence analysis (MCA), a descriptive/exploratory technique designed to analyze simple two-way and multi-way frequency, was used to identify biological profiles associated to SLN and ALND status [15], [16]. This representation aims to visualize the similarities and/or differences of profiles, simultaneously identifying those dimensions that contain the majority of the data variability. The positions of the points in the MCA graph are informative. Categories plotting close to each other are statistically related and are similar with regard to the pattern of relative frequencies and this association is statistically valuable (Lebart’s statistic) when the points are located far from the origin of the graph which represents a mean uninformative profile. SNL status detected by OSNA, BC subtypes, tumor size (T), histological grade (G), lymphovascular invasion (LVI) were introduced in the analysis as active variables whereas ALND status was introduced as supplementary variable. MCA provides a graphical representation of the active and supplementary variables projected on a plane formed by axes 1 and 2, which accounted for 67.6% of total variability, reproducing quite a significant percentage of the total chi-square value of the multi-way frequency table.

## Results

### Comparison between OSNA Assay and Histology

**Figure 1:**
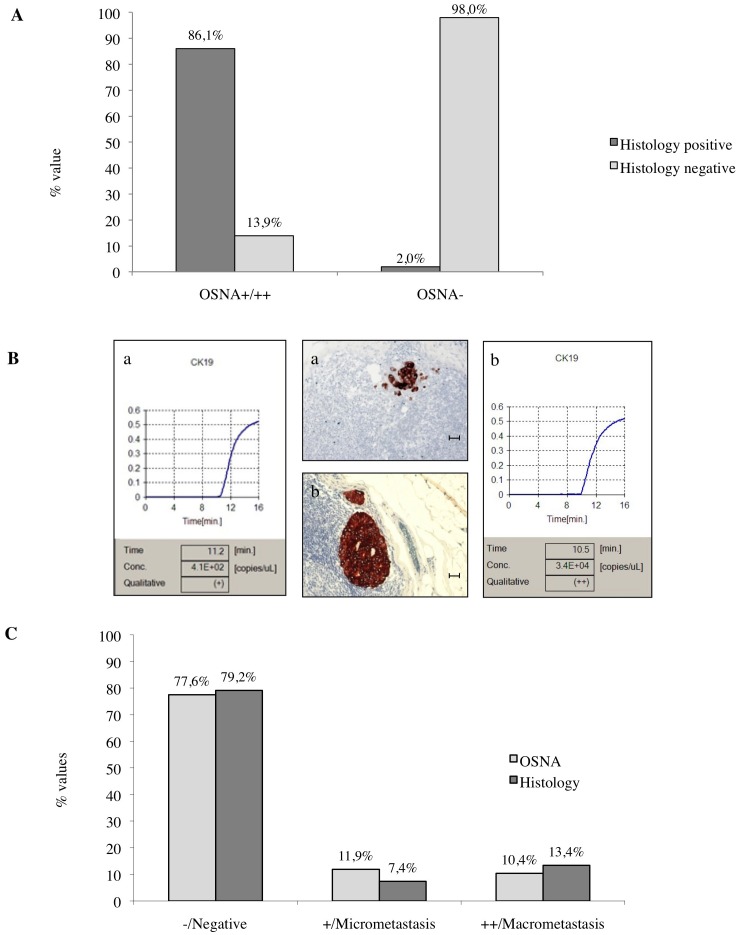
Correlation between OSNA assay and histology results in the entire series of 903 SLNs. *Panel A:* The histograms summarize the percentage of concordant and discordant results on SLNs analyzed by OSNA and histology. ***Panel B:*** Representative examples of CK19 immunostaining (immunoperoxidase) of a micrometastasis (a) and a macrometastasis (b) in two different SLNs compared to the matched OSNA curves (a-b) (scale bar = 90 µm). ***Panel C:*** The histograms show the distribution of −/negative, +/micrometastatic and ++/macrometastatic SLNs analyzed both by molecular and morphological methods. The percentage of micrometastases by OSNA was 11.9% and by histology 7.4%, whereas the percentage of macrometastases by OSNA was 10.4% and by histology 13.4%. Histology included H&E staining and IHC. OSNA−: <250; OSNA+ : >250≤5000; OSNA++: >5000 CK19 mRNA copies/µl.

In our series of 709 early BC patients, we analyzed a total of 903 SLNs, 1 in 535 (75%), 2 in 156 (22%) and more than 2 in 18 (3%) patients with an average of 1.3 SLNs per patient. All the SLNs were investigated both by intraoperative OSNA assay and by postoperative histological procedures (i.e. conventional H&E staining and IHC). The percentage of positive SLNs was 22.4% (202/903) by OSNA and 20.8% (188/903) by histological methods. Of the 709 early BC patients included in this study, 179 had at least one positive SLN. As summarized in Figure 1 panel A, 174 out of 202 (86.1%) and 687 out of 701 (98.0%) cases presented overlapping results by both molecular and histological methods. Among the 42 discordant cases, 28 (13.9%) were positive by OSNA and negative by histology whereas 14 (2.0%) were negative by OSNA and positive by histology. Of the 28 OSNA positive and histology negative SLNs, 25 were OSNA+ and 3 OSNA++. In the 14 OSNA negative and histology positive SLNs, we found 7 micrometastases and 7 macrometastases. Finally, IHC identified ITC in 6/687 SLNs (0.9%; data not shown). Taking into account the entire series of 903 SLNs included in our study, the percentage of micrometastases by OSNA (CK19 mRNA copies/µl >250≤5000) was 11.9% (108/903) and by histology 7.4% (67/903) whereas the percentage of macrometastases by OSNA (CK19 mRNA copies >5000) was 10.4% (94/903) and by histology 13.4% (121/903) (Figure 1 panel B-C). Whenever 2 or more SLNs from the same patient were tested by OSNA and showed different results (negative vs positive or+vs ++), the positive result or the one with the highest copy number was taken into account. The raw concordance between the OSNA and histological methods was 95%. Cohen’s Kappa statistic was equal to 86% [CI 95% (80–93)] with a statistically significant value (p<0.0001). By considering the histological procedures as the gold standard, in the entire series of 903 SLNs, the sensitivity of OSNA in detecting micrometastases and/or macrometastases was 93% [CI 95% (88–97)] and the specificity was 96% [CI 95% (94–98)]. The negative predictive value (NPV) was 98% [CI 95% (96–99)] and the positive predictive value (PPV) was 86% [CI 95% (79–91)]. In the 174 cases which were positive by both methods, a significant correlation was observed between the size of the metastases assessed by IHC and the CK19 mRNA copies detected by OSNA assay (Sperman’s coefficient: 0.827; p<0.0001).

### ALND Status Correlates to SLN Status Detected by OSNA

**Figure 2:**
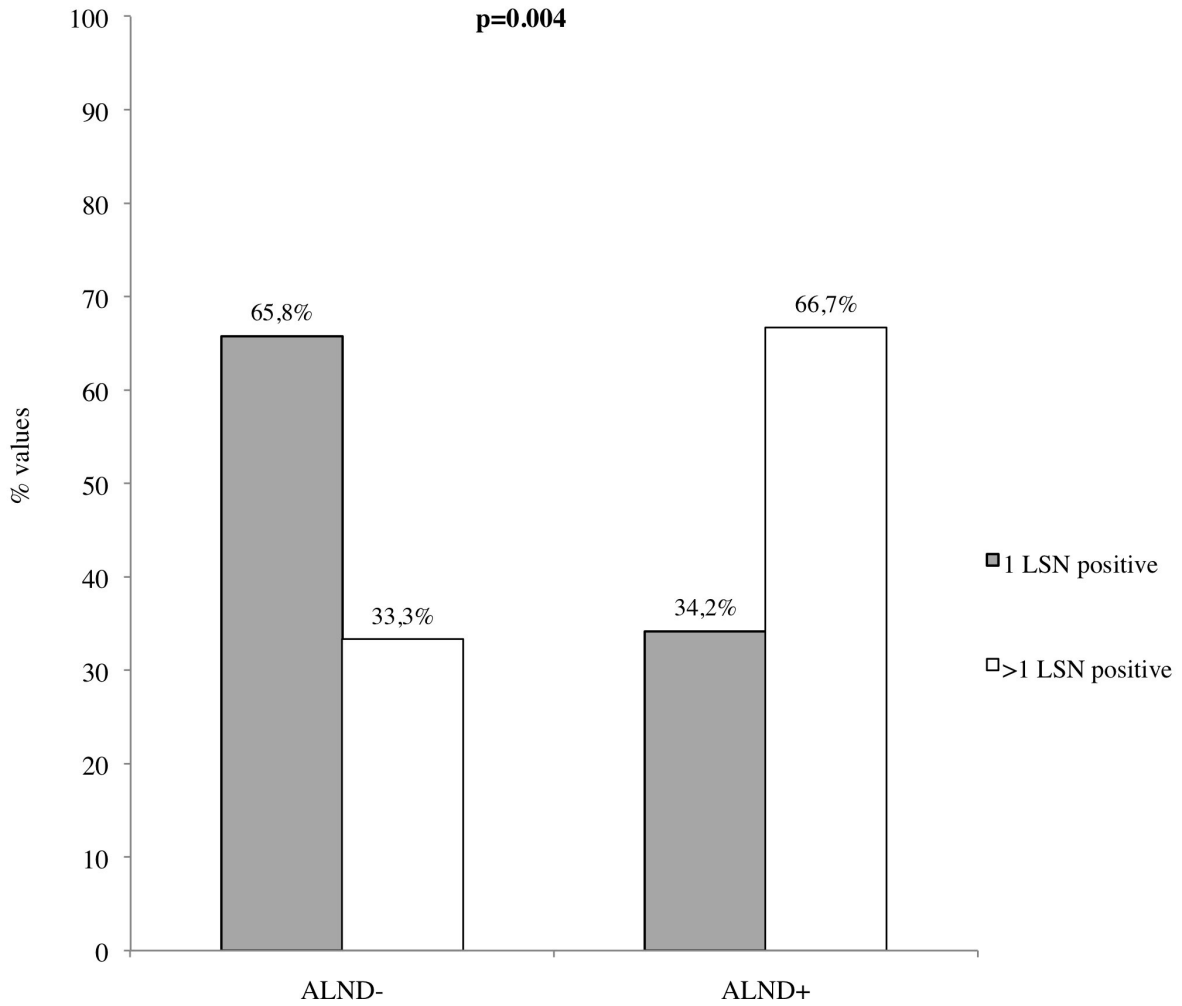
Relationship between number of SLNs tested by OSNA and ALND status. The percentage of negative ALND was significantly higher in patients with 1 OSNA positive SLN (65.8% vs 34.2%). Conversely, the percentage of positive ALND was significantly higher in patients with 2 or more OSNA positive SLNs (66.7% vs 33.3%) (p = 0.004).

Of the 179 patients with a positive OSNA assay, 111 (62%) had an ALND negative and 68 (38%) positive. Interestingly, of the 111 negative ALND, 72 (65%) were from patients with OSNA+ and 39 (35%) from patients OSNA++. Conversely, of the 68 positive ALND, 49 (72%) were from patients with macrometastasis (OSNA++) and only 19 (28%) from patients with micrometastasis (OSNA+). As shown in Table 2, in 118 out of 179 (66%) OSNA+/++ patients we tested 1 SLN whereas in 52 (29%) and in 9 (5%) patients we tested 2 or 3 or 4 SLNs respectively. Taking the CK19 mRNA copies into account, we found that of the 91 OSNA+ cases, 83 (91.2%) had at least 1 SLN positive of which 15 (18%) had also a positive ALND, 8 (8.8%) had 2 SLNs positive of which 4 (50%) had a positive ALND. In the group of 88 OSNA++ patients, we found 75 (85.2%) cases with 1 positive SLN of which 39 (44.3%) had a positive ALND and 13 cases (14.7%) with 2 or 3 positive SLNs of which 11 (78.5%) had a positive ALND. The percentage of negative ALND was significantly higher in patients with 1 OSNA positive SLN (65.8% *vs* 34.2%). Conversely, the percentage of positive ALND was significantly higher in patients with 2 or more OSNA positive SLNs (66.7% *vs* 33.3%) (p = 0.004) (Figure 2).

**Table 2:** Relationship between axillary non-sentinel lymph node status and number of SLNs analyzed.

Number of SLNs	OSNA+91		OSNA++88		Number of SLNs (%)
	57		61		
**1**	**ALND Negative**	**ALND Positive**	**ALND Negative**	**ALND Positive**	**118 (66%)**
	47 (+)	10 (+)	30 (++)	31 (++)	
	**28**		**24**		
	ALND Negative	ALND Positive	ALND Negative	ALND Positive	
	18 (−/+)	5 (−/+)	6 (−/++)	7 (−/++)	
**2**	**3 (+/+)**	**2 (+/+)**	**1 (+/++)**	**5 (+/++)**	**52 (29%)**
	−	−	1 (++/++)	4 (++/++)	
	**6**		**3**		
	**ALND Negative**	**ALND Positive**	**ALND Negative**	**ALND Positive**	
	3 (−/−/+)	−	1 (+/+/++)	1 (+/++/++)	
**3/4**	1 (−/+/+)	2 (−/+/+)	−	1 (−/−/−/++)	**9 (5%)**
Total	72	19	39	49	**179**

SLN: sentinel lymph node;

OSNA: one step nucleic acid amplification;

OSNA+: >250≤5000 cytokeratin 19 mRNA copies/µl; OSNA++: >5000 cytokeratin 19 mRNA copies/µl;

ALND: axillary lymph node dissection.

### Relationship between SLN Status and Bio-pathological Parameters

**Figure 3:**
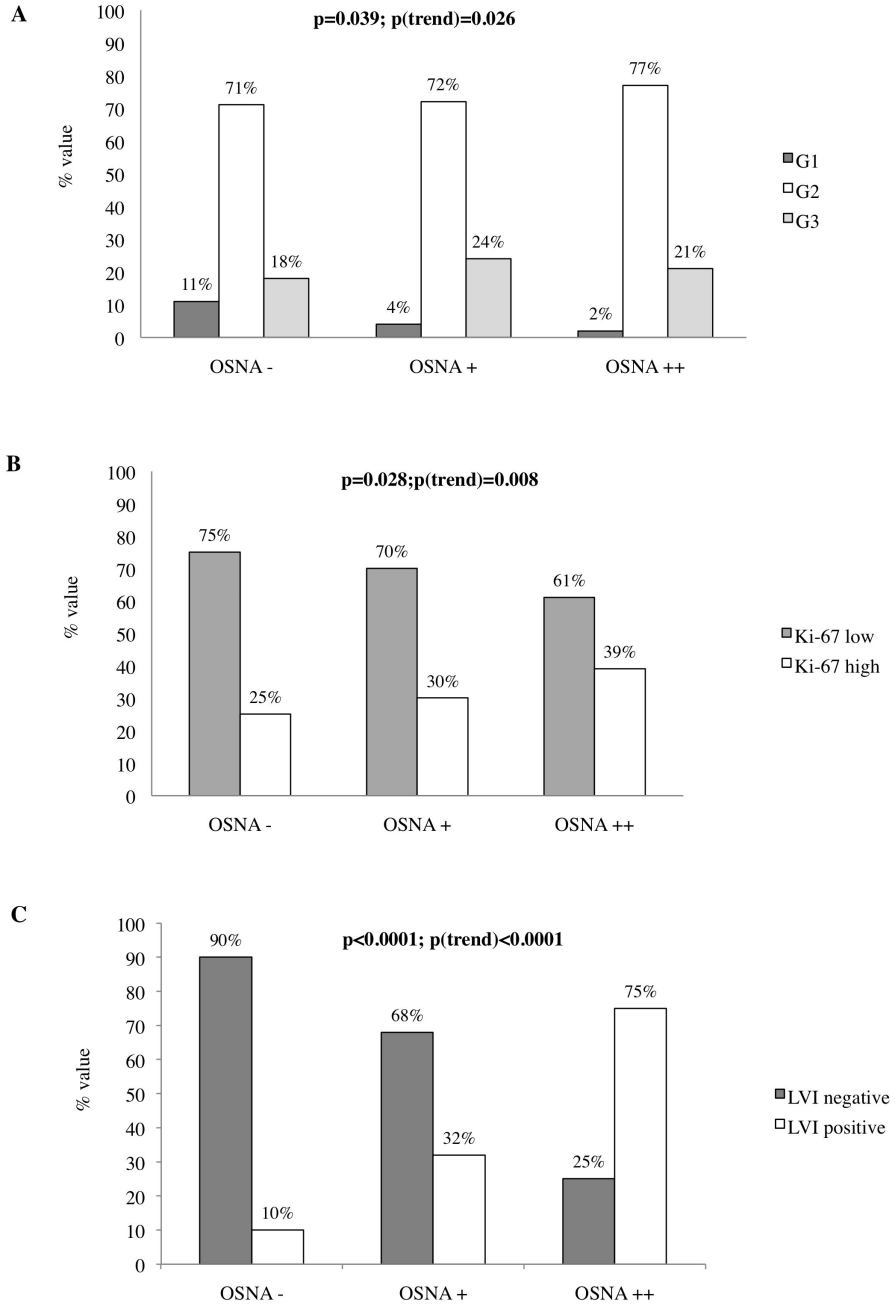
Relationship between SLN status and bio-pathological parameters. The histograms show that OSNA positive results are significantly correlated with ***(Panel A)*** poor differentiated tumor (p = 0.039), ***(Panel B)*** high proliferation index (p = 0.028) and ***(Panel C)*** presence of lymphovascular invasion (LVI p<0.0001).

**Figure 4:**
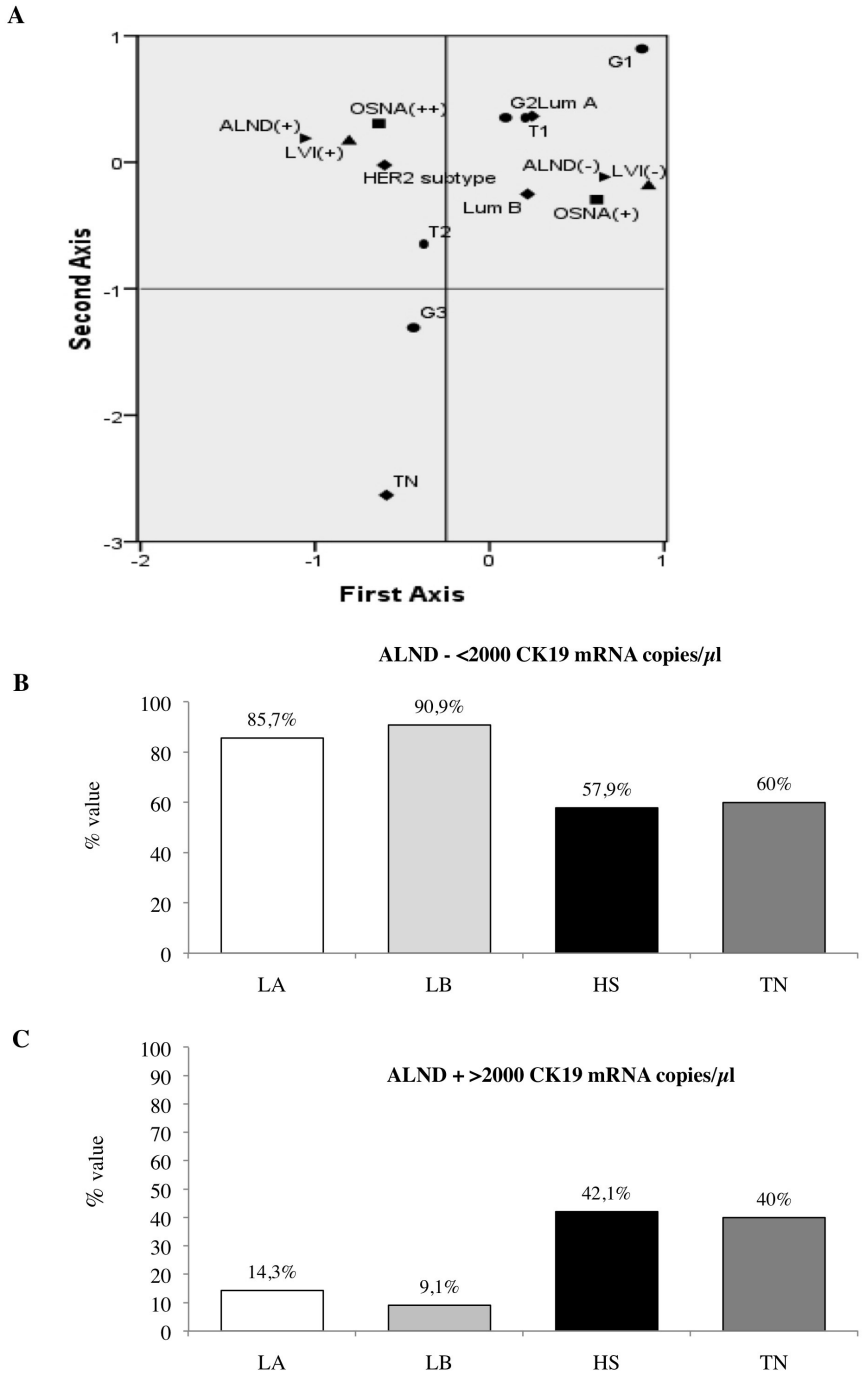
MCA of the 179 OSNA +/++ BC patients stratified by molecular subtypes and relationship between BC subtypes and ALND status in the subset of 91 OSNA+ BC patients. ***Panel A:*** The MCA graph demonstrates that ALND (+) is located in the quadrant containing the most aggressive phenotypes (T2 tumors, positive LVI, HER2 subtype, OSNA++) in contrast to ALND (−) which is associated to more favourable bio-pathological parameters (T1 tumors, absence of LVI, LA subtype). *LA: Luminal A; LB: Luminal B; HS: HER2 subtype; TN: Triple Negative ***Panel B :*** The histograms report the distribution of molecular subtypes in the 72 OSNA+ cases with ALND negative and CK19 mRNA <2000 copies/µl (range 270–1900). ***Panel C*** **:** The histograms report the distribution of molecular subtypes in the 19 OSNA+ cases with ALND positive and CK19 mRNA >2000 copies/µl (range 2100–4600).

Figure 3 shows that OSNA positive results were significantly correlated with (Panel A) poor differentiated tumor (p = 0.039), (Panel B) high proliferation index (p = 0.028) and (Panel C) presence of lymphovascular invasion (LVI p<0.0001). Of interest, all these correlations progressively and significantly increased from patients with negative to patients with macrometastatic SLNs (p(trend) for G = 0.026, for Ki-67 index = 0.008, for LVI <0.0001). No significant correlations were observed between SLN status detected by OSNA and the other conventional bio-pathological parameters analyzed (data not shown). In order to better investigate the relationship between SLN status detected by OSNA, ALND status and conventional bio-pathological factors, we stratified our series of 647 invasive BC by molecular classification. We found that 428 BC (66.2%) were LA, 65 (10%) were LB, 108 (16.7%) were HS, and 46 (7.1%) were TN. We used MCA to study the complex interrelationships among pathological (T, G, LVI), and biological variables (ER, PgR, HER2, Ki-67) the latter clustered into phenotypic subtypes (LA, LB, HS, TN) visualizing their link with SLN and ALND status. As illustrated in Figure 4 panel A along the first axis, the test demonstrates the contrast between T2 tumors, positive LVI, HER2 subtype, OSNA++ (upper left quadrant) and T1 tumors, negative LVI, Luminal subtype, OSNA+ (upper right quadrant). In order to obtain an indication of the predictive value of the graphical configuration produced by MCA, we added ALND status as a supplementary variable. Interestingly, the 2 categories (ALND+*vs* ALND −) are located in opposite quadrants (upper left quadrant ALND+*vs* upper right quadrant ALND −) contrasting the 2 groups previously described; such location of the ALND status in the graph may identify bio-pathological profiles as potential predictive factors of ALND positivity, providing the basis to investigate their predictive role using regression analysis. Focusing on the 91 OSNA+ BC patients, we observed that all the 72 cases with negative ALND presented a CK19 mRNA copy number <2000 (range 270–1900) regardless of BC molecular subtypes (Figure 3 panel B). Conversely, all the 19 cases with positive ALND had a CK19 mRNA copy number >2000 (range 2100–4600) (Figure 3 panel C). Of interest, nearly 88% of luminal BC with a CK19 mRNA copy number <2000 had an ALND negative.

### Logistic Regression Analysis

The following variables were studied by logistic regression analysis: BC subtypes, LVI, tumor grading, tumor size and CK19 mRNA copies/µl. As summarized in Table 3, the model indicates that the probability of finding an ALND positive is significantly higher in patients with HER2 positive tumors (OR 2.82, 95% CI 1.33–6.00; p = 0.007), with LVI positive (OR 58.85, 95% CI 17.10–200.32; p<0.0001) and with OSNA++ SLNs (OR 4.76, 95% CI 2.47–9.19; p<0.0001).

**Table 3:** Logistic regression analysis of negative factors predictive of ALND involvement.

Probability of a positive ALND			
	OR	95%CI	p-value
**Subtypes**			
LA *vs* LB	1.16	0.45–3,01	0.755
**LA ** ***vs*** ** HS**	**2.82**	**1.33–6.00**	**0.007**
LA *vs* TN	1.56	0.46–5.28	0.476
**LVI**			
**Negative ** ***vs*** ** Positive**	**58.5**	**17.10–20.32**	**<0.0001**
**Grading**			
G1+G2 *vs* G3	1.66	0.82–3.89	0.162
**T**			
T1 *vs* T2	1.52	0.81–2.85	0.191
**OSNA+ ** ***vs*** ** OSNA++**	**4.76**	**2.47–9.19**	**<0.0001**

ALND: axillary lymph node dissection; OR: odd ration; CI: confidence interval; LA: Luminal A; LB: Luminal B; HS: HER2 subtype; TN: Triple Negative; LVI: lymphovascular invasion; OSNA: one step nucleic acid amplification; OSNA+: >250≤5000 cytokeratinn 19 mRNA copies/µl; OSNA++: >5000 cytokeratin 19 mRNA copies/µl.

### Analysis of Discordant Cases

In our series there were 42 discordant cases, which were all sampled from patients for whom a single SLN was analyzed by OSNA. The 28 SLNs positive by OSNA and negative by histology and the 14 SLNs negative by OSNA and positive by histology (discordance rate 4.65%) were investigated in more detail as reported in Table 4. In the first group, 25 out of 28 morphologically negative SLNs were OSNA+ (89.3%) and 3 OSNA++ (10.7%). Only 3 out of the 25 (12%) OSNA+ SLNs presented a positive ALND whereas all 3 OSNA++ cases had a positive ALND. Interestingly, the copy number of the 6 SLNs (3 OSNA+ and 3 OSNA++) with a positive ALND were consistently higher (OSNA+ ≥2500≤3200 and OSNA++ ≥8000≤21000) than the copy number detected in the remaining 19 SLNs presenting a negative ALND (≥270≤1700). Concerning BC subtypes, we found that 40% of HS (2/5) and only 6.6% of LA tumors (1/15) presented a positive ALND. To find out to what extent these discrepancies may be influenced by a sampling bias, the 28 OSNA positive and morphologically negative SLNs were cut into further levels at an interval of 50 µm until no remnants remained. After this extended analysis, we did not detect metastatic deposits in any of the evaluated samples. Postoperative IHC detected metastases, up to 3 mm in diameter, in 14 OSNA negative patients who underwent ALND in a second surgery. In detail, of the 14 cases investigated, 7 were micrometastases (0.1–1 mm) and 7 were intranodal metastases (2.5–3.0 mm) by histology. All the latter 7 cases were LA BC with a negative ALND, and a negligible LVI. Otherwise, of the 7 macrometastatic cases, 2 (28.5%) had a positive ALND with the primary tumor presenting a significant LVI.

**Table 4:** Analysis of the 42 discordant cases between OSNA and histology.

Number of SLNs	Size of metastasis	OSNA Results	OSNA Copies/µl	Molecular Subtype	ALND Results
**Histology Results: negative**					
					
2	−	+	≥2500≤3200	HS	**+**
1	−	+	3600	LA	**+**
3	−	+	≥6300≤1700	HS	−
3	−	+	≥300≤540	LB	−
2	−	+	≥270≤830	TN	−
14	−	+	≥280≤1400	LA	−
3	−	++	≥8000≤21000	LA	**+**
**Total 28**					
**Histology Results: micrometastasis**					
7	0.1–1 mm	−	<250	LA	−
**Histology Results: macrometastasis**					
2	2.5 mm	−	<250	LA	**+**
2	3.0 mm	−	<250	LA	−
3	2.5–3 mm	−	<250	HS	−
**Total 14**					

OSNA: one step nucleic acid amplification; SLN: sentinel lymph node; HS: HER2 subtype; LA: Luminal A; LB: Luminal B; TN: Triple Negative; ALND: axillary lymph node dissection.

## Discussion

More than 20 studies, including a wide range of BC patients, have been published in the last five years which show the reliability of the molecular OSNA assay in detecting metastases in the SLN. The first set of papers consists of the early works whose goal was to develop, debug and validate the OSNA method [8], [17]–[19], testing both SLN and/or non-SLN; a second one consists of studies in which the authors, analyzing one half of the SLN by OSNA, compared molecular results with histology which represented the gold standard [20]–[22]. A third set of papers consists of studies in which the whole or almost whole SLN was analyzed by OSNA in an aim to verify the reliability of the method compared to standard procedures conducted on historical or randomized prospective cohorts of patients [23]–[30]. The present study is part of the second group of papers in which one half of SLN was investigated by OSNA assay comparing results to standard morphological procedures. Nevertheless, unlike other studies, we took our analysis one step further and we assessed the risk of a positive ALND in relation to CK19 mRNA copy number in the SLN. Concomitantly, we considered the latter parameters in the context of the molecular classification of BC. Our series consisted of 903 SLNs sampled from 709 consecutive BC patients and, to the best of our knowledge, this is one of the largest prospective series in which patients with both micrometastases (+) and macrometastases (++), detected by OSNA assay, underwent immediate ALND. In our consecutive series of BC the concordance rate between OSNA and histology was 95% and, by considering the histological procedures as the gold standard, the sensitivity and specificity of OSNA assay in detecting SLN metastases was 93% and 96% respectively. In contrast to other authors [17], [20], our concordance analysis did not exclude discordant cases mainly due to tissue allocation bias. Nonetheless, the concordance rate between OSNA and histology reported in our series resembles other studies. In the prospective multicenter study by Snook and colleagues [20], which included intraoperative examination of SLN from 204 BC patients, the overall concordance rate between OSNA and histopathology was 96% with a sensitivity of 91.7% and a specificity of 96.9%. Overlapping results were reported by Bernet and collegues [31] and by Le Frère-Belda et al [22]. Also the two studies [24], [27] which intraoperatively examined the whole SLN by OSNA except for a 1 mm thick central slice of the lymph node, arrived at the conclusion that OSNA is an accurate assay which could significantly reduce the need for second surgery. Starting from the hypothesis that combining histological and molecular assessment of metastases on the same SLN might not fully reproduce the actual load of cancer cells present in the SLN and may create problems in decisions regarding axillary dissection, several recently published studies analyzed the whole SLN by OSNA comparing results with those obtained on historical cohort [26], [28], [29] or randomizing patients to receive OSNA assay or conventional histological procedures [25], [31], [32]. In both cases authors concluded that OSNA makes it possible to standardize SLN analysis and it is clinically useful for immediate decisions regarding axillary dissection. In our large prospective series of 709 BC patients, the concomitant analysis of SLN by OSNA and by histology may allow to verify that the rate of micrometastasis was higher by molecular assay than by standard histology. These findings are in line with studies [25] which analyzed the entire SLN by OSNA and confirmed that this difference may be a reflection of the higher sensitivity of the molecular method compared to histology. Our OSNA positive patients were submitted to ALND independent from the presence of micrometastasis (+) or macrometastasis (++). Of the 179 OSNA positive BC patients, 61 had more than 1 SLN tested. As expected, we evidenced that the probability of having a subsequent positive ALND was significantly higher when more than 1 SLN was positive. We delved deeper into the matter and we examined the likelihood of a positive ALND according to the levels of OSNA positivity (OSNA+ *vs* OSNA++) taking concomitantly into account the different, molecularly distinct, BC subtypes (10,12). First, we evaluated the association between ALND positivity, conventional pathological factors and BC subtypes by multiple correspondence analysis (MCA) [15], [16], an alternative method for analyzing multiple categorical variables by graphically visualizing their interrelationships. MCA showed that OSNA++ and ALND+presented a significant dispersion around the origin and were located in the same quadrant associated to aggressive tumor phenotypes such as HER2 subtype, presence of LVI and larger tumor size. These findings strongly support the correlation between OSNA++ and ALND positivity. MCA associations were statistically supported by multivariate Cox regression analysis in which both HER2 subtype, LVI and presence of macrometastasis by OSNA are independent predictor factors of ALND positivity. Our findings, in line with Ohi et al. [30], demonstrated that the OSNA assay significantly predicts ALND positivity mainly when associated to unfavourable bio-pathological parameters. Furthermore, focusing on OSNA+ SLNs, we showed, for the first time, that the concomitance of a low CK19 mRNA copy number (<2000) and a luminal tumor phenotype may be a useful, objective tool for predicting non-SLN positivity. In agreement with other studies which analyzed SLN both by OSNA and histology [8], [17], [19], [20], we found a discordant rate between the two methods of 4.7%. Although the tissue allocation bias is one of the main causes of discordance, another cause, as already reported [25], [32], is the higher sensitivity of OSNA in detecting micrometastases. In this context, our data are in agreement with other authors since, among the 42 discordant cases, 25 (60%) were OSNA+/histology negative. Of interest, in this series we found that the two cases with a positive ALND presented a OSNA copy number >2500 whereas the remaining 23 cases, with a negative ALND, had a OSNA copy number <2000. Starting from this observation it would be of particular clinical relevance to define a cut-off value which is capable of accurately identifying the subset of patients bearing a micrometastatic SLN to be selected for therapeutic ALND. This is an important issue because the clinical impact of SLN micrometastasis continues to be an area of great debate [33], [34]. The availability of a molecular method which provides less subjective and quantitative results may be a useful tool in this context. To date, all the breast nomograms [35]–[38] which have been set up to predict the presence of metastatic spread in non-SLN are based on standard histology and have a number of limitations mainly due to the difficulties in assigning the discriminating size of tumor load. Therefore, the choice of a further axillary treatment for a SLN with minimal involvement, mainly for small BC (≤2 cm) of low-intermediate grade, remains largely a decision between the surgeon and the patient.

The OSNA assay, together with a presurgical analysis of tumor subtype, could represent a valid and objective tool to set up a novel model capable of more accurately predicting axillary involvement. Omission of ALND could be proposed in patients with a micrometastatic SLN with a low CK19 mRNA copy number (<2000) and luminal tumor phenotype. To validate these findings, the predictive value of CK19 mRNA copy number is currently under investigation on a new prospective series of BC patients treated in our Institute in whom the whole SLN is analyzed by the molecular OSNA method.
